# Policy interventions to improve health through housing: A scoping review

**DOI:** 10.1371/journal.pone.0358689

**Published:** 2026-09-29

**Authors:** Anne L. Buffardi, Paul Pascall Jones, Lucien Begault

**Affiliations:** 1 London Borough of Islington, London, United Kingdom; 2 Department of Primary Care and Population Health, University College London, London, United Kingdom; U of U: University of Utah, UNITED STATES OF AMERICA

## Abstract

Despite decades of research documenting the effects of poor housing on health, there is much less evidence of the effectiveness of different housing interventions to improve health outcomes. Moreover, existing research tends to focus on a specific population group or aspect of housing. This scoping review aimed to identify the range of policy instruments and interventions that could improve health by improving housing quality, stability, affordability and availability, with a focus on interventions being implemented in practice, including those not published in the academic literature. For each intervention, we documented aims, effects, actor roles, cost, housing domain, target population, city and country. Based on 37 evidence reviews and reports, we identified 327 unique interventions, with economic and social regulation (e.g., regulating pricing and private rented sector practices, changing planning processes) and direct or contracted provision (e.g., providing and upgrading social housing) much more common than other policy instruments. Evidence on effectiveness varied considerably, with more outcomes reported for physical modifications, economic regulation and tax policy, and limited evidence on social housing, public information, social regulation, effects on health outcomes and measures along multiple stages of a change pathway. Thirty-two types of actors were involved in authorising, financing, implementing and/or enforcing interventions, spanning public and private sectors and levels of government. Intervention costs were infrequently stated and varied substantially, with multiple reviews noting the mismatch between those who finance, implement, benefit and save. This scoping review uncovered more interventions and a wider range of policy instruments than identified in previous systematic reviews, suggesting an important and underutilised role for the grey literature in identifying existing programmes and highlighting evidence gaps. Taking advantage of these natural experiments, embedding more robust evaluation into current interventions and comparing the effectiveness of different policies that aim to achieve similar outcomes offer the opportunity for research to better inform housing policy and maximise its potential to improve health.

## Introduction

The influence of wider determinants on physical and mental health has been discussed for decades [1,2] and highlighted as a key pillar of population health [3]. Since the early 2000s, housing as an important wider determinant of health – particularly the harmful effects of poor quality and insecure housing – has received increasing attention from scholars and decision-makers alike. This heightened awareness is reflected in World Health Organisation guidelines [4] and recently in the United Kingdom in the 2021 Charter for Social Housing Residents, Social Housing (Regulation) Act 2023, Renters’ Rights Act 2025 and Awaab’s Law, named after a 2-year-old boy who died from a severe respiratory condition due to household mould.

Scholars have characterized four main pathways through which housing influences health: quality, stability, affordability and broader neighbourhood factors, noting the more developed evidence base for the former two [5,6]. A burgeoning set of conceptual frameworks has attempted to illustrate the interconnections between housing, neighbourhood contexts, effect pathways and a range of health outcomes [5,7–12]. For example, Bentley and colleagues illustrate the direct effects of home environments with hazards on injury rates and homes with mould on the incidence of asthma and in turn, morbidity and mortality rates, health-adjusted life years and health inequalities [11]. Rolfe and colleagues’ realist theoretical framework identifies mechanisms through which neighbourhood and housing quality, services and support could affect tenant health and wellbeing by influencing their levels of stress, relaxation, sense of status and opportunities for socialization [9].

Empirically, poor quality housing, including cold temperatures, damp, mould and poor air quality is associated with respiratory conditions [13–25]. Overcrowding has been linked to increased rates of tuberculosis [26], higher risk of future illness, including respiratory diseases [27], heart disease [28,29], stomach cancer [30] and poor mental health outcomes [31]. Children in particular experience increased stress [32,33], poor sleep [32], and behavioural issues [5,18,32,34,35]. Cold exposure, fuel poverty and stress related to insecure or unstable housing have been associated with changes in blood pressure, cardiovascular risks, decline in self-reported health [5,16,17,36–38] and increases in anxiety and depression [5,7,39–41]. Conversely, homeownership, reflecting greater housing stability, is associated with higher levels of self-reported mental and general health [42–46]. Housing cost burden is linked to depressive symptoms, particularly for renters [47], and has been critiqued for exacerbating neighbourhood segregation and health inequalities [26,40,44,48–52]. Studies have also investigated outcomes for specific population groups, including migrants and refugees [53] and people experiencing homelessness [53–58].

Relative to the evidence demonstrating the influence of housing on physical and mental health, fewer studies assess the effects of specific interventions to address housing quality, stability and affordability. A 2025 systematic overview of reviews only identified six reviews which reported intervention effects on health [59]. Half of these reviews focused on improvements to informal settlements [60–62] and one on interventions to prevent malaria [63]. The remaining two reviews were broader, covering physical modification, rental assistance, relocation [64,65] and urban regeneration interventions [65], but not the full range of policy instruments that governments and other actors can deploy to influence housing, including economic and social regulation and direct provision of housing.

In addition to being fewer in number, the quality of intervention studies is often weak, despite drawing predominantly or exclusively on the academic literature. There are a small number of trials [66] and robust natural experiment evaluations [67–69]. Nonetheless, systematic reviews have characterised the evidence base on the effectiveness of housing interventions on health outcomes as mixed, noting the absence and limitations of conducting large-scale trials, measuring longer-term effects on health status [40,44,65,66,70,71] and incorporating economic evaluation [72].

Increasingly, scholars have called for greater use of complex systems approaches that better account for the interaction between housing and welfare policies, market dynamics and spillover effects of one actor’s behaviour on another [7,8,15,39,54,73,74]. At the same time, Rutter and colleagues argue that “emerging complex systems approaches to public health are only rarely operationalised in ways that generate relevant evidence or effective policies” [74] (p.2602). This observation has led to calls for pragmatic pluralism [75,76] and a more balanced approach between three types of evidence: causal evidence on the effects of exposures, intervention evidence on sets of actions needed to modify complex systems and implementation evidence on the conditions necessary to carry out these changes [76,77].

This scoping review aims to help bridge these gaps, with a focus on intervention and implementation evidence. Scoping studies seek to characterise the breadth and depth of evidence and clarify the conceptual boundaries of a topic area in order to identify gaps and determine if a full systematic review is warranted. This review looks at a broad range of interventions across housing domains and population groups that capture the complex housing system, asking four questions. First, what interventions exist or have been proposed that could improve housing quality, stability or affordability? Second, what is the evidence of their effectiveness on health and other reported outcomes? Third, what are the distinct roles that different actors and institutions play? And finally, what are the costs and estimated cost savings? We pay particular attention to the role of local government, given where public health teams in England are based and discussion of the potential of local government to influence wider determinants of health, including housing [2,8].

## Methods

We followed the 6-stage methodological framework for scoping studies, which involves: identifying the research questions (as noted above), identifying relevant articles, study selection, charting the data, collating, summarising and reporting results and consultation [78,79].

### Identifying relevant studies

We identified studies through evidence reviews conducted by the UK Collaborative Centre for Housing Evidence (CaCHE) and Local Government Association (LGA) in May-June 2025. CaCHE reviews, all of which focused on housing, were identified through the search term ‘review’. LGA reports were identified through the topic filter ‘housing, planning and homelessness’. These searches were also supplemented with reviews and policy reports circulated through five cross-sector and local government policy and practitioner networks from September 2024 to June 2025.

Responding to our research question focused on existing or proposed policies, this identification strategy sought to uncover intervention and implementation evidence that may not be published in academic journals. This was intended to complement existing systematic reviews of reviews of the academic literature that have dominated the evidence base, to maximise the relevance and use of this review for decision makers and practitioners and to identify evidence gaps that scholars could help fill.

CaCHE reports were more comprehensive reviews, predominantly of the academic literature, and provided more detail about their methods. LGA reports were by definition specific to local government, although many discussed the role of other actors as well, particularly central government. The level of detail on their review methods varied, but many involved interviews, stakeholder roundtables and case examples from local governments, which provided more detail on the implementation of interventions than other sources. Reports circulated through policy and practice networks tended to emphasise needs and problems, provide a topline description of interventions, and in some cases made recommendations that appeared to be normative positions rather than based on evaluations of existing interventions. These reports often covered multiple types of interventions, where CACHE and LGA reviews typically focused on a specific policy instrument (e.g., tax policy), housing tenure (e.g., private rented sector) or population group (e.g., older adults, asylum seekers).

### Study selection

Articles were eligible for inclusion if they were evidence reviews or reports, published in English, that reviewed at least one specific intervention that intended to improve housing quality, stability or affordability. Some reviews also included interventions to address housing availability, so we expanded our inclusion criteria and housing domain category to include availability. We did not place restrictions on geography, the type of effects reported or how outcomes related to health were defined, instead documenting and classifying existing evidence to clarify the boundaries of this topic area, as scoping reviews are intended to do. We therefore included studies reporting any type of effect, including but not limited to health or a specific definition of health, and subsequently categorised type of effect in the data charting stage. We excluded reviews whose primary focus was on healthy places or cities, which covered a much broader remit beyond housing, often including environmental, transport, active travel, play and leisure, food and community development interventions. We also excluded reports that were focused on a niche issue or locale (e.g., Dublin-Belfast economic corridor), resident experiences, behaviours and tenant participation or that related to Covid.

### Charting the data

Unlike literature reviews, scoping studies provide an analytical reinterpretation of the information [78], which took place in part during this stage. For each evidence review, we first extracted and coded each specific housing intervention mentioned. We excluded more general recommendations or guidance, often related to the importance of more or better data, improved coordination, integration and partnership working and early engagement. We also excluded interventions that were not primarily housing-related, such as those that sought to increase employment and earnings of households in need of affordable housing.

Drawing on Salamon’s 2002 typology [80], we classified the full list of housing interventions into 7 broad policy instruments: direct or contracted goods and services, social regulation, public information, grants, loans, fiscal policy and economic regulation, and other financing schemes and arrangements (e.g., joint ventures). These categories cover the range of policy types included in more recent umbrella reviews of public health, place-based policies and macroeconomic determinants of health inequalities [81–83]. In addition to these broad policy instruments, we also grouped specific interventions into general intervention types to provide an overview of the range of interventions within each broad instrument. For example, fiscal policy and economic regulation included regulating borrowing, regulating pricing, changing tax policy and managing settlement and payment processes, among others.

For each specific intervention, we documented: the housing domain (quality, stability, affordability and/or availability), intervention effects, effect type (health status, self-reported/perceived health, health or social care use, relational, property conditions, economic, housing market, process, outputs, other), type of evidence and the intervention cost. The data dictionary provides explanations and examples for each effect type. For example, health status covers both physical and mental health and includes specific conditions (e.g., asthma), injuries, life expectancy and functional ability. Self-reported/perceived health covers physical, mental and general health, as reported by study respondents, perceived quality of life, wellbeing and safety. General health is a self-reported question in surveys designed by the UK Office for National Statistics which asks respondents to rate on a 5-point scale from very good to very bad ‘How is your health in general?’ Health or social care use includes hospital admissions, falls requiring medical attention and hours of home care.

The data charting file also includes specific intervention aims, target populations, city/region and country where interventions were implemented, as well as a short data extract and reference details of original source articles to enable more detailed searches.

We coded the involvement of actors according to 4 roles: authorizing/legislating, financing, implementing/administering and enforcing. We used consistent decision rules to identify which roles apply to each policy instrument to distinguish roles that may not be applicable to a particular instrument from roles where the actor information was missing. For example, regulation should always have an actor in an authorisation role, whereas grants should always have actors in financing and implementing roles. When the data extract from the review article did not specify actor roles, we updated roles in three circumstances, based on supplementary information: when the intervention was a named programme (e.g., Disabled Facilities Grant, Warm Homes programme), using information provided on their websites; for interventions where actor information was included in another data extract for the same specific intervention in the same country (e.g., Renters Rights Bill in the UK); and when interventions reflected duties that are the responsibility of a specific level of government (e.g., central government authorising changes to Capital Gains Tax and local authorities authorising local planning processes). The data charting file includes columns for the original, explicitly stated actor roles or lack thereof, as well as columns for updated roles based on this supplementary information, to enable decision-makers and practitioners to identify potential interventions they could use.

More than one third (n = 14) of review articles were coded by two team members to check for inter-rater reliability, address differences and update the data dictionary and coding as needed. The subsequent classification and analysis of specific variables, such as general intervention type and actor roles, provided an additional opportunity to quality assure and update original codes for consistency.

### Collating, summarising and reporting results

For key variables linked to each of our four overarching questions, we calculated descriptive statistics or a qualitative thematic analysis, which is presented in the results section. We present findings as a proportion of: all reported interventions (n = 412), unique interventions (n = 327) and interventions reporting any type of effect (n = 132). Table 1 (frequency of policy instrument and general intervention type) and text presenting frequency of housing domains use unique interventions as the denominator, since multiple studies report on the same intervention. Table 2 (types of effects) presents the proportion of interventions reporting any type of effect. Table 3 (type of effect by policy instrument) and text presenting effectiveness of interventions and costs use all interventions as the denominator, since different studies reporting on the same intervention may report different effects or costs. Table 4 (actor roles) also draws from all reported interventions for the same reason and presents the total number of times a specific actor was involved in a specific role.

**Table 1:** Frequency of policy instrument and general intervention type.

	Count	% of unique interventions
**Direct or contracted provision of goods and services** ^1^	91	28%
Implement physical modifications	27	8%
Provide legal and alternative dispute resolution (ADR) services	18	6%
Provide temporary accommodation	10	3%
Provide affordable housing	8	2%
Provide services for tenants	6	2%
Provide supported housing	6	2%
**Fiscal policy and economic regulation** ^2^	84	26%
Regulate pricing, including private rented sector rent, Local Housing Allowance and temporary accommodation	29	9%
Use/change tax policies	14	4%
Regulate land release, assembly, pricing, options	8	2%
Manage settlement and payment processes	7	2%
Compulsory purchases by local authorities	7	2%
Regulate borrowing	5	2%
**Social regulation** ^3^	81	25%
Regulate private rented sector practices, including protection of tenants’ rights, health and safety, licensing, landlord registration, length of rental agreements, empty homes and energy efficiency	28	9%
Use/change local authority planning processes	25	8%
Change/update standards or policy	11	4%
Establish new standards	10	3%
**Grants**	44	13%
Including grants for physical modifications, affordable housing, regeneration and homelessness services		
**Loans**	15	5%
Including loans for physical modifications and regeneration and establishing loan funds for property purchase and affordable housing		
**Other financing schemes and arrangements**,	8	2%
Including establishing joint ventures and property funds, nominations agreements and property shared ownership		
**Public information**	4	1%
Including advice to households, homeowners and private rented sector landlords		

^1^Other direct and contracted provision of goods and services (n = 16; 5%) included providing supported housing, safety equipment, services for homelessness, empty homes, home maintenance, and landlords, and managing assets.

^2^Other fiscal policy and economic regulation interventions (n = 14; 4%) included regulating receipts, providing guarantee, deposit and insurance schemes, establishing leasing and letting schemes and establishing a local authority owned housing company.

^3^Other social regulation (n = 7; 2%) included standards specific to energy efficiency and enforcing existing standards or policy.

**Table 2:** Types of effects reported.

	Count	% interventions reporting any effects
Health-related outcomes		
Health status	24	18%
Self-reported health	24	18%
Health or social care use	8	6%
Other intermediate outcomes		
Housing market	41	31%
Economic	28	21%
Property conditions	23	17%
Relational	3	2%
Outputs, processes & satisfaction		
Outputs	26	20%
Improved processes	8	6%
User satisfaction	5	4%
Other^1^	13	10%

Note: More than one third of interventions reporting any effects mentioned more than one type of effect (n = 47/132; 36%), reflected in the count total.

^1^Other effects included anti-social behaviour, substance misuse, maintenance levels, management standards and reputation.

**Table 3:** Types of effects across policy instruments.

	Health related		Other intermediate outcomes		Outputs, processes & satisfaction		Not stated or applicable		Total
Direct or contracted provision of goods & services	31	8%	15	4%	13	3%	67	16%	126
Grants	4	1%	2	0.5%	4	1%	36	9%	46
Social regulation	4	1%	12	3%	5	1%	77	19%	98
Fiscal policy and economic regulation	2	0.5%	33	8%	1	0.2%	79	19%	115
Loans	0	0%	1	0.2%	4	1%	10	2%	15
Other financing schemes & arrangements	0	0%	0	0%	0	0%	8	2%	8
Public information	0	0%	0	0%	1	0.2%	3	1%	4
Total	41	10%	63	15%	28	7%	280	68%	412

**Table 4:** Roles of different actors in housing interventions.

Actor type^1^	Authorising	Financing	Implementing	Enforcing
Local government	35	130	262	33
Central government	147	79	37	2
Private rented sector (PRS) landlords		23	37	
Developers		11	27	
Tribunals and courts			6	15
Housing associations	1	3	17	
Ombudsman services			2	14
Private contractors			15	
Private sector investors		10	3	
Alternative dispute resolution (ADR) services			12	
Energy providers		5	5	
Voluntary and community sector organisations			8	
Private rented sector (PRS) tenants		8		
Homebuyers		3	3	

Note: Across all records (n = 412), unstated actor roles were updated based on supplementary information for 42 (10%) interventions regarding authorising roles, 133 (32%) regarding financing roles, 117 (28%) regarding implementing roles and 30 (7%) regarding enforcing roles. Both original and updated actor roles are included in the Data Charting File. Table totals do not sum to 412 because not all roles are applicable to all policy instruments, more than one actor type may be involved in the same role, particularly implementation, and infrequently mentioned actor types listed below are not presented in the table.

^1^Other, less frequently mentioned actor types included The Royal Society for the Prevention of Accidents, registered housing providers, regional government, private managing agents, private lenders, Trusts, Ofgem, the National Lottery, Mayor of London, Local Government Association, Care and Repair and Home Improvement agencies, homeowners and health care providers.

The Preferred Reporting Items for Systematic Reviews and Meta-Analyses Extension for Scoping Reviews (PRISMA-ScR) checklist is included as a supplemental file (S1 Checklist).

### Consultation

Finally, we consulted intended audiences of this scoping review at three phases. Early in the process of identifying relevant studies and types of housing interventions, we had discussions with 7 public health and housing professionals working for a large research consortium and in local, regional and central government roles. After the initial scoping and analysis was completed, we shared preliminary findings and a draft version of the data charting file with three public health housing experts whose research assesses effectiveness of government interventions, who provided insights on drivers of variation in practice and useful feedback on potential uses of the data and gaps this resource helps to fill, additional variables and how to make the data charting file more accessible and easier to search. We then shared thematic data extracts (e.g., interventions related to temporary accommodation) and an updated version of the data charting file with 10 local, regional and national practitioners and scholars to test its relevance and utility and identify potential next steps.

## Results

Of the 231 articles identified, 37 (16%) evidence reviews and reports met the inclusion criteria and were included in this scoping study (Fig 1). Based on the 37 evidence reviews, we identified 412 total interventions and 327 unique interventions aimed at improving housing quality, stability, affordability and/or availability.

**Fig 1:**
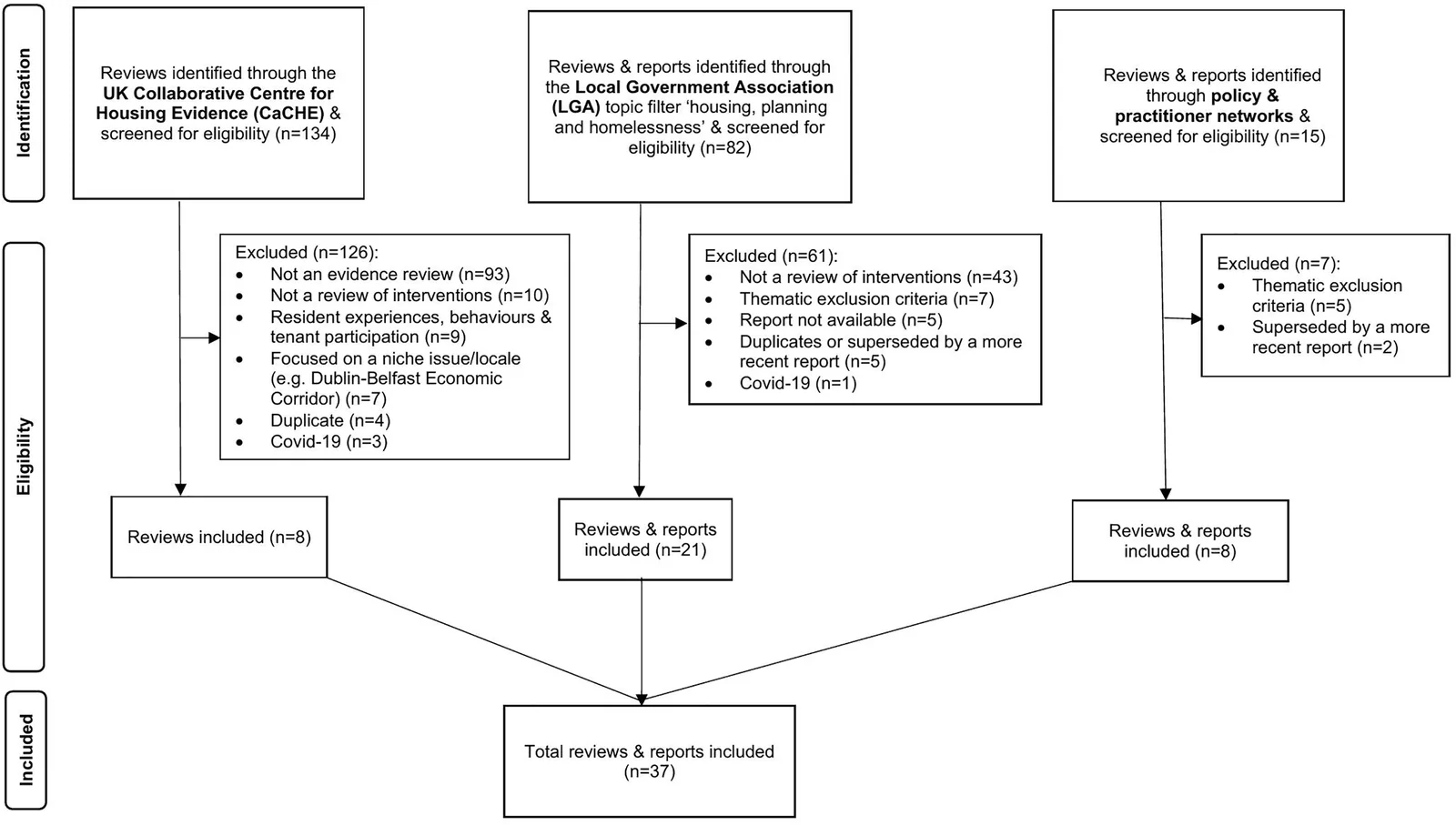
PRISMA flowchart.

### Policy instruments and housing interventions

Across the 7 broad policy instruments, direct or contracted provision of goods and services (n = 91; 28%) and fiscal policy and economic regulation (n = 84; 26%) were most common, followed by social regulation (n = 81; 25%). Grants (n = 44; 13%), loans (n = 15; 5%), other financing schemes and arrangements (n = 8; 2%) and public information (n = 4; 1%) featured much less frequently.

Different policy instruments can be used to achieve the same intended outcome. For example, improving the affordability of housing can be influenced by building new social housing with government funds (direct provision), regulating rent prices or price increases by private sector landlords, regulating the proportion of homes built by private developers that meet a price threshold and by changing tax policy (fiscal policy and economic regulation of different types of actors). These policy instruments vary in the extent to which they are visible, direct, coercive and can be implemented through existing institutions (referred to as automaticity) [80]. These instrument attributes may guide the choice of one over the other and can help to explain variation in policy choices across different administrations and political contexts.

The 327 unique housing interventions fell into 53 general intervention types, of which 41 (77%) were mentioned by more than one review article, indicating substantial consistency across the broad set of interventions that could be considered. Table 1 illustrates the range of intervention types across policy instruments. Some interventions reflect statutory duties, such as local government obligations to provide temporary accommodation for people experiencing homelessness. Other interventions are discretionary, licensing of private rented sector properties, for instance. Regulating pricing (n = 29; 9%), regulating private rented sector practices (n = 28; 9%), implementing physical modifications (n = 27; 8%) and using or changing local authority planning processes (n = 25; 8%) were the most commonly raised general interventions. Providing legal and alternative dispute resolution services also represented a relatively high proportion (n = 18; 6%); however, the vast majority (15 of 18) came from a single review article.

Of the four housing domains, which broadly reflect overarching aims, quality was most common (n = 127; 31%) and stability was least common (n = 81; 20%) among unique interventions. One-fifth of unique interventions (n = 65; 20%) sought to address more than one domain; for instance, restricting rent price increases aims to improve affordability as well as tenant stability. However, there are also examples of trade-offs between domains, namely the availability and affordability of housing.

### Effectiveness of housing interventions

Overall, the strength of the evidence base supporting the interventions identified here varied considerably, as did the types and sources of evidence that the review articles drew on to present the interventions, implementation contexts and effects. The reviews included evaluations, surveys, case studies, statistical analyses, modelling, expert consultation, government policy guidance and evidence reviews; unfortunately, the level of variation and methodological detail means that we are not able to calculate the proportion of interventions that could be considered to have been robustly evaluated.

Notably, two-thirds (n = 280; 68%) of interventions included in the review articles did not report effects, although many noted intended aims. In some cases, this is because the interventions had not yet been enacted or implemented when the review article was submitted, like the Renters Rights Bill. For others, information about outcomes may have been in original source articles that the evidence review referenced but did not specify. However, there were instances where interventions were proposed to address an evidenced need or problem, but the effectiveness of that specific intervention was not itself reported or cited.

Moreover, where effects were reported, they reflected different types of outcomes and units of analysis, examining changes for individuals, households, properties, neighbourhoods and the housing market, including investor, landlord and homebuyer behaviours. In some cases, evaluations reported outputs, such as service delivery or participation rates, rather than outcomes, or reported user satisfaction or improved processes, like faster dispute case resolution.

Table 2 presents this heterogenous range among the interventions that reported any type of outcome, output or change. More than one third (n = 47; 36%) reported more than one type of effect. Review articles provided information about three types of health-related outcomes: health status (e.g., injuries, respiratory conditions), self-reported health and changes in health or social care use. However, as the table illustrates, many reported effects were more proximate, such as improvements in property conditions, and examined areas beyond health. Effects related to the housing market were most common and included house price volatility, rate of rent increases, tenancy turnover and residential mobility and segregation.

Looking across policy instruments, more health-related outcomes were reported for direct or contracted provision of goods and services, albeit still only representing 8% of all interventions (31/412) and one quarter of the types of effects reported for this policy instrument (31/126) (Table 3). Health-related outcomes were also reported for 9% of grant interventions (4/46), 4% of social regulation interventions (4/98) and 2% of fiscal policy and economic regulation interventions (2/115). No health effects were reported for loans, public information or other financing schemes.

Other intermediate outcomes were more common for fiscal policy and economic regulation (n = 33; 8% of all interventions; 29% of effects for this policy instrument).

Of the interventions that did report effects, the greatest proportion related to physical modifications of housing units, implemented through direct provision (n = 33; 25%), grants (n = 8; 6%) and loans (n = 2; 2%), with strong, albeit variable, evidence on health conditions [84–89]. For example, there is moderate to strong evidence that modifications to improve warmth and energy efficiency improves asthma symptoms, respiratory health and self-reported general health of adults and children and low certainty evidence on effects on mental health and health outcomes of older adults [86].

Evidence on the impact of regulating of private rented sector practices, such as licensing, tenants’ rights, and landlord registration, suggests that better control and enforcement of standards related to quality and stability of tenure is associated with improved mental health outcomes [39,68,85,90], and no evidence that this regulation has resulted in landlords leaving the private rented sector [91]. Some evidence indicates that reducing the frequency of rent increases leads to a decrease in tenant turnover, increases length of residency for vulnerable populations and ethnic minorities and contributes towards a shift in homeownership [92–95]. There is also evidence that rent control policies can lead to unintended outcomes, such as poorer quality homes in rent-controlled areas, increased rent in decontrolled areas and reduced mobility [92,96–100]

Legal and alternative dispute resolution services, such as mediation schemes between private renters and landlords, were associated with avoiding more burdensome, stressful and costly court processes [101,102], achieving speedier resolutions [101,103] and high levels (62–75%) of out of court settlement [101]. Establishing new standards, such as the Decent Homes Standard for social housing, and associated investments has reduced the number of non-decent homes [88]. However, these evaluations of dispute resolution and improvements in housing quality did not study subsequent effects of these interim changes on the health of household members. Evidence on the few examples of loan schemes was mixed and similarly, only reported interim outcomes [88,104].

In terms of effects of tax policies on the housing market, there is strong evidence that higher stamp duty reduces transactions, house prices and residential mobility [105]. Local government tax increases on empty homes do not appear to be a sufficient deterrent, with a 10% increase in empty homes across the UK since 2018 despite these measures [104].

The evidence base on the effectiveness of social housing, public information, local government planning processes and other types of social regulation appears to be the least developed or referenced in evidence reviews. Overall, there were few reports of either unexpected or unintended negative consequences of specific interventions, aside from those mentioned above. Nor were there examples comparing different types of interventions that sought to achieve the same outcome, as in the affordability example discussed at the outset.

### Institutional and actor roles

Evidence reviews mentioned the involvement of 32 types of institutions and actors, including local government, central government, housing associations, energy providers, tribunals and courts, government regulatory agencies, the independent Housing Ombudsman Service, private landlords, investors, developers, contractors and managing agents, homebuyers, homeowners and tenants (Table 4). Relative to the public and private sectors, the reported involvement of voluntary and community sector (VCS) organisations in any capacity was much less frequent. In some cases, the actor type was non-specific, with reviews not distinguishing between different types of social housing providers, for instance. Where a role was applicable to an intervention, information was not stated for 37% (82/224) of authorising roles, 59% (150/233) of financing roles, 40% (163/412) of implementing roles, and 70% (35/50) of enforcing roles, before supplementary information was incorporated.

The range of actors and institutions involved in housing interventions thus covers both public and private sectors as well as different levels of government. It also reflects a wide variety of professional disciplines, from planners, chartered surveyors and lawyers to social workers, tax specialists and economists.

Actor roles broadly corresponded to policy instruments and general intervention types, with central government typically responsible for authorising and financing interventions and local government implementing and enforcing them. The use of local government planning processes for social and economic regulation and local government funds for direct and contracted provision of housing and services are notable exceptions to these patterns. Private sector actors were most often involved in financing and implementing roles.

Sixty-four percent (n = 119) of unique interventions involved more than one actor type, for instance, central government authorising regulations, private landlords responsible for implementing them and local government for enforcement. In some cases, multiple actors had shared responsibilities for the same role: grants, loans or joint ventures between developers, investors, central government and/or local government to finance new housing or invest in regeneration.

### Costs and return on investment of housing interventions

The costs of specific interventions were infrequently stated, a significant limitation in the evidence base. Of the 69 (17%) of reported interventions where financial information was provided, some presented costs per housing unit or funding recipient, whereas other reports considered costs alongside cost savings or return on investment calculations. Each is relevant for policy recommendations to be actionable. For example, even if an intervention provides a positive return on investment, if the upfront cost exceeds the budget available, it is not a financially feasible intervention.

Costs were reported much more often for grants, compared to all other policy instruments and more often for interventions that involved physical modifications. Although the costs associated with regulation may be less visible than those required to provide goods and services directly, they are not without cost, namely staff time, including for enforcement.

The level of investment needed to implement different interventions varied substantially, as did the scale. For example, the 2024 Disabled Facilities Grant programme covers adaptations ranging from less than £5,000 to £30,000, in aggregate approximately £700 million a year. Some individual interventions were relatively low cost, hundreds of pounds for a physical modification, whereas other programmes invested tens of billions of pounds: an estimated £22 billion of central government funds and £15 billion from social housing providers for the Decent Homes Programme between 2000 and 2011 [106].

Costs were rarely provided relative to an overall budget or other investments. An exception is two related reviews comparing costs of different physical modifications to improve housing quality, which ranged from approximately £635 per dwelling for modifications to address excess heat, carbon monoxide or ergonomics to over £20,000 per household to address overcrowding [107,108]. Given different publication years, prices are not directly comparable, although could be converted to be. Evidence reviews did not discuss cost thresholds (floors or ceilings) and potential economies of scale. Multiple reviews did however note the mismatch between those who finance, implement, benefit and save as a result of different interventions, which is a critical consideration and potential factor limiting the implementation of effective housing interventions.

### Evidence gaps

As highlighted in the previous three subsections, there are clear evidence gaps. Table 5 highlights areas where there is a particular lack of evidence where future research and analyses should be directed. Most notable are gaps on intervention effectiveness and outcomes and on intervention costs and savings, linked to our second and fourth research questions, respectively.

**Table 5:** Evidence gaps.

**Effectiveness**
Intervention outcomes, including health status and measures along multiple stages of a change pathway
Effectiveness of interventions to improve housing affordability
Comparison of different policy instruments that seek to achieve similar aims (e.g., improve affordability or quality), including benefit-cost analyses within the same institution
**Implementation processes**
Intervention costs
Specific actor roles in authorising, financing, implementing and enforcing interventions
**Context**
Variation in implementation & effectiveness across contexts and actor types

Evidence of intervention effectiveness could be strengthened in three ways, by: measuring changes along multiple stages in a longer pathway of change, evaluating interventions to improve housing affordability and comparing different policy instruments that seek to achieve similar aims. When intervention effects were reported, they were often limited to shorter-term, proximate changes. The evidence base would benefit from more comprehensive assessments that track intervention activities and outputs through to interim outcomes, unintended consequences, effects on health conditions and population-level inequalities, particularly for interventions with less direct causal pathways. Existing conceptual frameworks offer a structure through which this work could be undertaken [5,7–12].

More interventions were focused on housing quality than other domains and evidence for these outcomes was stronger. This pattern is likely partly driven by methodological reasons, since physical modifications to improve quality represent a discrete intervention and direct pathway of influence. Arguably, however, affordability considerations underlie housing quality and stability. Therefore, despite greater methodological challenges, more research on interventions to address affordability is warranted.

Our review found no examples where different policy instruments or interventions that aim to achieve a similar aim (e.g., improve quality, stability or affordability) were compared to one another. Scholars have noted the methodological, research funding and publication drivers which explain why individual studies and evidence reviews focus on one type of instrument or subset of interventions [73,74,109]. In practice, however, comparisons across instruments and interventions are taking place, implicitly or explicitly, when decision-makers are allocating fixed resources. More transparent, robust comparisons, including cost-effectiveness and benefit-cost analyses would be a substantial contribution to the evidence base. Furthermore, until pooled budgets and substantive financial transfers between institutions are feasible (e.g., investing NHS savings to build or improve social housing), estimating costs and savings within the same institution (e.g., investing savings in local authority Adult Social Care costs into local authority housing interventions) will provide decision-makers with more actionable evidence.

In terms of evidence gaps related to implementation processes, specifying key implementation considerations – costs and the roles of different actors – would similarly help to identify what policy instruments and interventions are financially feasible and within whose control to take action. Of all the categories we reviewed, costs were the largest omission. While actor roles were more commonly included or could be identified through supplementary sources, there were particular gaps in specifying which actors are financing and implementing direct or contracted provision of goods and services and who is responsible for authorising, implementing and enforcing regulations.

Our review identified evidence from 18 countries, 113 subnational geographic and administrative units (states, regions, cities, boroughs) and 1 international region and 32 types of institutions and actors. However, this variation was rarely exploited to better understand what instruments, interventions and actor configurations may be more feasible to deliver and more effective for different population groups in different contexts.

## Discussion

This scoping review sought to characterise the breadth and depth of evidence on housing policies that have the potential to affect health, identifying i) interventions that could improve housing quality, stability or affordability, ii) evidence on their effectiveness, iii) actor roles and iv) costs. It bridges housing and health disciplines and research, policy and practice by more explicitly incorporating policy typologies and investigating key implementation considerations for research recommendations to be actionable. This is intended to better balance the literature, which has thus far been heavily oriented towards downstream health effects of exposures, with insufficient attention to the range of interventions and aspects of implementation that may be required to address housing conditions that, in turn, could affect health.

The review offers unique contributions to our first research question, highlighting the large number and wide range of existing and proposed interventions that could improve housing quality, stability and affordability. Our search yielded more interventions than in previous systematic reviews, suggesting an important and underutilised role for grey literature in identifying intervention and implementation evidence. For example, we identify examples of fiscal policies and economic regulation that have largely been absent from the public health literature to date but could be potentially influential levers to improve housing-related health conditions. Sixteen European countries have some type of rent stabilisation policy [110], but there is little discussion of rent regulation in England, relative to calls to improve the quality of and build more social housing [85].

Our review also helps to identify interventions implemented in the past that no longer receive funding, such as the Decent Homes Programme. It captures interventions that have been tried with limited effectiveness, including additional taxes on empty homes and some previous loan schemes, which can help to inform alternative approaches or programme redesign. Furthermore, it distinguishes among different types of policy instruments which can be used to address the same intended outcome – direct provision, grants, loans and regulation to conduct physical modifications to improve housing quality, for instance.

The review also starts to build a more systematic overview of our third research question on actor roles, clarifying what policy levers are within whose control and the dependencies that exist between levels of government and public and private sectors. For public health teams, this information can be used to identify other actors, both within (e.g., planning departments) and outside of local government, with whom they will need to collaborate to address this wider determinant of health. The multi-actor nature of many interventions can also help to explain implementation delays and variation.

There are notable evidence gaps that the review surfaces. In particular, the review provides limited evidence to answer our second and fourth research questions on the effectiveness of interventions, both overall and specifically related to health, as well as on intervention costs and savings. These gaps are influenced in part by study limitations, discussed below, and can be used to guide future research.

Our review highlights opportunities to better embed evaluation into existing programmes to understand how implementation varies across contexts and population groups and crucially, how interventions affect health. It also suggests ways that the grey and academic literatures could better be sequenced to inform one another. For example, the grey literature could be more substantively included in evidence reviews to identify an initial set of interventions and direct future academic research, focused on longer-term effects in order to guide decisions about targeting and expansion. The grey literature could then be subsequently used to better understand variation in implementation at scale, including evidence from specific locales in a geographically or politically bounded jurisdiction. Both the grey and academic literatures currently lack the level of detail on actor roles and costs needed for decision-makers, an important consideration for future work. Understanding how costs and savings are unevenly distributed across actors may help to generate alternative financing options and move discussions beyond repeated calls for some actors to invest in housing improvements (e.g., social and private sector landlords), while savings accrue to others (e.g., NHS). Recent legislation in England offers a valuable opportunity to put these changes into practice by conducting comparative process and outcome evaluations of these natural experiments, including unintended consequences as resources are reallocated from some housing interventions to others.

As new legislation is enacted and new programmes created, the list of policy interventions will continue to expand and therefore never be complete. Nevertheless, the number and range of interventions identified here suggests that a systematic review or set of linked reviews is warranted, that span policy instrument types and that sufficiently capture interventions being implemented in practice which are not published in the academic literature. As AI tools continue to improve, they offer additional opportunities to quickly identify a range of interventions being proposed and implemented. However, scoping reviews offer transparent search and selection criteria, critical appraisal and analytical reinterpretation of existing information, as well as a publicly available data charting file for others to use, expand and refine.

### Limitations

Our review is limited in several ways. It does not include evidence from the academic literature that was not referenced in evidence reviews. In addition, more economic evaluations of current interventions may exist, but may be published in economics journals, which may not be picked up in evidence reviews focused on health or housing. Our selection of evidence reviews, therefore, may help to explain why reviews of the majority of specific interventions did not report evidence on outcomes, costs or savings.

The more rapid nature of reports in the grey literature may preclude measurement along multiple stages in a longer pathway of change and of more distal outcomes. An absent or less stringent peer review process may not require these types of outcomes as a condition of publication. These characteristics reflect longstanding critiques of the grey literature and the range of intended purposes, audiences, report formats and level of detail that they provide [111]. Furthermore, scoping reviews do not systematically assess study quality, so we are unable to calculate the proportion of studies that were weak, moderate or strong to confirm or refute this concern about the variable quality of the grey literature.

Linked to our aim for the findings to be relevant to public health teams, the evidence reviews we included are more heavily oriented towards local government interventions in England. Many LGA reports mentioned the role of other actors, particularly central government. CaCHE reviews included evidence from other regions and countries, predominantly Scotland, the United States, Canada, Australia and other European countries, but all were high income countries. Therefore, the review does not reflect the full range of interventions being implemented or proposed, particularly in low- and middle-income countries.

As noted, based on the evidence we assessed, this scoping review is unable to draw substantive conclusions about the effects and cost of most interventions, and to a certain extent, actor roles. It does, however, uncover these evidence gaps and provides a data charting file on 327 interventions, which can be expanded and refined as these gaps started to be filled.

## Conclusion

Public health has a long history of conducting needs assessments and documenting harms to people’s physical and mental health. This scoping review aims to enable the field to direct a comparable level of attention in identifying, implementing and evaluating interventions to address the needs and negative health effects that have been so well articulated. Moreover, it intends to do so in a way that is relevant for decision-makers who must meet statutory responsibilities and allocate fixed resources across a vast array of options. With an increased focus on housing as an influential wider determinant of health, this study offers a timely contribution to help advance both evidence and action.

## Supporting information

S1 Checklist PRISMA extension for Scoping Reviews (PRISMA-ScR) checklist. (DOCX)

S2 Data Data charting file. (XLSX)
