# Supplementary material for: Policy interventions to improve health through housing: A scoping review
Source: PLoS One. 2026 Sep 29;21(9):e0358689. doi: 10.1371/journal.pone.0358689 (PMC13623142; doi:10.1371/journal.pone.0358689)
Supplement: Supplementary file 2 [file pone.0358689.s004.docx]

We thank the reviewers for their detailed and helpful questions and recommendations and the editor for the very useful clarification on revisions and reconciling reviewers’ comments.

We have revised the abstract, introduction, methods, results and discussion sections and added 2 new tables to the manuscript. We have also updated the data dictionary and added 6 new columns to the data charting file: 1) effect type, 2) ‘other’ effects and explicitly stated actor roles in 3) authorizing, 4) financing, 5) implementing and 6) enforcing.

Below we provide a point-by-point response to each comment.

Required for acceptance

1. **Operationalise "health" and stratify by outcome type**. Health is never defined, and the Results slide between intervention completion, housing change, and health outcomes (Reviewer 2). The Methods must specify what counts as a health endpoint, and the Results must distinguish interventions reporting health endpoints from those reporting only intermediate measures. A layered framework, signposting where evidence stops, would address this without requiring the retitle Reviewer 2 suggests.

We have undertaken additional analyses to distinguish between types of reported effects, adding two new columns to the data charting file for effect type and effect ‘other’ and provided operational definitions and examples of each effect type in the updated data dictionary.

In the introduction section, we have specified that existing evidence demonstrates the influence of housing on *physical and mental* health, reflecting the associations presented in the third paragraph. In addition to citing existing conceptual frameworks, we have given examples of two in the text: ‘A burgeoning set of conceptual frameworks has attempted to illustrate the interconnections between housing, neighbourhood contexts, effect pathways and a range of health outcomes [5,7–12]. *For example, Bentley and colleagues illustrate the direct effects of home environments with hazards on injury rates and homes with mould on the incidence of asthma and in turn, morbidity and mortality rates, health-adjusted life years and health inequalities [11]. Rolfe and colleagues’ realist theoretical framework identifies mechanisms through which neighbourhood and housing quality, services and support could affect tenant health and wellbeing by influencing their levels of stress, relaxation, sense of status and opportunities for socialization [9].*’

In the methods section, we have added:

- Study selection subsection: ‘*We did not place restrictions on geography, the type of effects reported or how outcomes related to health were defined, instead documenting and classifying existing evidence to clarify the boundaries of this topic area, as scoping reviews are intended to do.*
- Charting the data subsection: ‘For each specific intervention, we documented: the housing domain (quality, stability, affordability and/or availability), intervention *effects, effect type (health status, self-reported/perceived health, health or social care use, relational, property conditions, economic, housing market, process, outputs, other)*, type of evidence and the intervention cost.

In the results section, we have:

- Updated the ‘Effectiveness of housing interventions’ subsection: adding a new table (2: Types of effects reported) and provided additional explanation in the text: ‘Moreover, where effects were reported, they reflected different types of outcomes and units of analysis, *examining changes for individuals, households, properties, neighbourhoods and the housing market, including investor, landlord and homebuyer behaviours. In some cases, evaluations reported outputs, such as service delivery or participation rates, rather than outcomes, or reported user satisfaction or improved processes, like faster dispute case resolution. Table 2 presents the heterogenous range among the interventions that reported any type of outcome, output or change. More than one third (n=47; 36%) reported more than one type of effect. Review articles provided information about three types of health-related outcomes: health status (e.g. injuries, respiratory conditions), self-reported health and changes in health or social care use. However, as the table illustrates, many reported effects were more proximate, such as improvements in property conditions, and examined areas beyond health. Effects related to the housing market were most common and included house price volatility, rate of rent increases, tenancy turnover and residential mobility and segregation*.’
- Added a new ‘Evidence gaps’ subsection, including a new table highlighting key gaps related to effectiveness, implementation processes and context, and six paragraphs of text discussing these gaps.

2. **Tighten inclusion criteria and the scope of claims**. State the source-level information required for an intervention to be included and analysed. Many reports lack the granularity to support claims about effectiveness, cost, or actor roles. Two specific implications: (a) the actor-role imputation flagged by Reviewer 1 (lines 203–204) should be reported as missing rather than filled by decision rule; and (b) the use of health-system framing to support housing interventions is a worthwhile research direction but one this corpus cannot adequately answer, and should be framed carefully or deferred to future work.

Inclusion criteria. The methods section indicates *‘Articles were eligible for inclusion if they were evidence reviews or reports, published in English, that reviewed at least one specific intervention that intended to improve housing quality, stability or affordability.’* As noted above, ‘*We did not place restrictions on geography, the type of effects reported or how outcomes related to health were defined….*’

Actor roles. We have added four new columns to the data charting file with original actor roles, conducted additional analyses and QA and clarified our approach and rationale in the methods section:

*‘We used consistent decision rules to identify which roles apply to each policy instrument to distinguish roles that may not be applicable to a particular instrument from roles where the actor information was missing. For example, regulation should always have an actor in an authorisation role, whereas grants should always have actors in financing and implementing roles. When the data extract from the review article did not specify actor roles, we updated roles in three circumstances, based on supplementary information: when the intervention was a named programme (e.g. Disabled Facilities Grant, Warm Homes programme), using information provided on their websites; for interventions where actor information was included in another data extract for the same specific intervention in the same country (e.g. Renters Rights Bill in the UK); and when interventions reflected duties that are the responsibility of a specific level of government (e.g. central government authorising changes to Capital Gains Tax and local authorities authorising local planning processes). The data charting file includes columns for the original, explicitly stated actor roles or lack thereof, as well as columns for updated roles based on this supplementary information, to enable decision-makers and practitioners to identify potential interventions they could use.’*

Scope of claims. We have made the limited information on health effects more explicit in the:

- Abstract: ‘limited evidence on social housing, public information, and social regulation, *effects on health outcomes and measures along multiple stages of a change pathway*’
- ‘Effectiveness of housing interventions’ subsection and new ‘Evidence gaps ‘subsection, as noted above
- Discussion section: ‘*There are notable evidence gaps that the review surfaces. In particular, the review provides limited evidence to answer our second and fourth research questions on the effectiveness of interventions, both overall and specifically related to health, as well as on intervention costs and savings*.’
- Limitations section: ‘*As noted, based on the evidence we assessed, this scoping review is unable to draw substantive conclusions about the effects and cost of most interventions, and to a certain extent, actor roles*.’

3. **Reflect critically on grey literature bias**. The 68% no-outcome figure is partly an artifact of source selection (Reviewer 2). Limitations should address this and indicate how future research might use grey literature leads to retrieve effect evidence from academic databases.

We have been more explicit on grey literature bias in the discussion and limitations sections: ‘*These gaps are influenced in part by study limitations, as discussed below, and can be used to guide future research. …* ‘*Our selection of evidence reviews, therefore, may help to explain why reviews of the majority of specific interventions did not report evidence on outcomes, costs or savings. The more rapid nature of reports in the grey literature may preclude measurement along multiple stages in a longer pathway of change and of more distal outcomes. An absent or less stringent peer review process may not require these types of outcomes as a condition of publication. These characteristics reflect longstanding critiques of the grey literature and the range of intended purposes, audiences, report formats and level of detail that they provide [116]. Furthermore, scoping reviews do not systematically assess study quality, so we are unable to calculate the proportion of studies that were weak, moderate or strong to confirm or refute this concern about the variable quality of the grey literature.’*

In the discussion section, we have added ‘*It also suggests ways that the grey and academic literatures could better be sequenced to inform one another. For example, the grey literature could be more substantively included in evidence reviews to identify an initial set of interventions and direct future academic research, focused on longer-term effects in order to guide decisions about targeting and expansion. The grey literature could then be subsequently used to better understand variation in implementation at scale, including evidence from specific locales in a geographically or politically bounded jurisdiction. Both the grey and academic literatures currently lack the level of detail on actor roles and costs needed for decision-makers, an important consideration for future work.*’

Recommended

4. **An evidence gaps table (R1) and a cost-summary table (R2)** would strengthen the resource value.

As noted, we have added a new evidence gaps table. We reviewed the information on costs again and based on the limited (18% of interventions – the most incomplete of all of our data charting categories) and variable types of financial information (more so than intervention effects), we have not added a new cost summary table. In our judgment, a table would give it more prominence than the data warrants. We have made that limitation clear in the abstract, results and discussion sections.

5. **Number the four research questions and reference them through the Results; justify the locale exclusion more fully (R1).**

We have numbered the research question and refer to them in the results and discussion sections.

We have also added more information about the geographic breadth the scoping review covers ‘*evidence from 18 countries, 113 subnational geographic and administrative units (states, regions, cities, boroughs) and 1 international region’* and clarified the statement in the methods section to ‘We have also excluded reports that were focused on a *niche issue or locale (e.g. Dublin-Belfast economic corridor)*’. This example refers to a report about spatial planning policy following the Brexit referendum and the Northern Ireland protocol that is unlikely to be broadly applicable. We have also suggested in the discussion section that in the future, the grey literature could help ‘*to better understand variation in implementation at scale, including evidence from specific locales in a geographically or politically bounded jurisdiction*.’

6. **One framing point. AI tools** can now generate intervention inventories quickly but cannot guarantee provenance, defensible framing, or practitioner relevance. The distinctive value of a scoping review like this lies in curation, judgment, and the auditable data charting file — a sentence to this effect in the Discussion might help readers locate the contribution.

We have added to the discussion section: ‘*As AI tools continue to improve, they offer additional opportunities to quickly identify a range of interventions being proposed and implemented. However, scoping reviews offer transparent search and selection criteria, critical appraisal and analytical reinterpretation of existing information, as well as a publicly available data charting file for others to use, expand and refine*.’

**Reconciling the reviews**. Reviewer 1's points are worth addressing as written. Reviewer 2's framework critique should be taken seriously but does not require retitling; the required changes above are necessary.

**Reviewer #1**: Thank you for the opportunity to review this paper, which is a helpful scoping review of housing interventions to improve public health. Overall, I think the idea and implementation of this project are strong, but I have a few methodological concerns along with some small suggestions on paper presentation. Please see full comments below.

Abstract:
• Abstract is clear

Introduction:
• Helpful framing
• **Typo, line 126: should be “determine IF a full systematic review is warranted”**

Thank you for catching this typo, which we have corrected.

• **Would be helpful to number the four questions so they can be easily referenced later**
We have numbered the questions, as suggested.

**Methods:**
**• It seems like there would be a lot of evidence from specific locales, so it’s unclear to me why these were excluded from the current review. Can the authors provide more justification for this decision? Why is this a review of reviews rather than an overall review of the evidence?**

As noted in the response to the Editor above, we have added more information about the geographic breadth the scoping review covers ‘*evidence from 18 countries, 113 subnational geographic and administrative units (states, regions, cities, boroughs) and 1 international region’* and clarified the statement in the methods section to ‘We have also excluded reports that were focused on a *niche issue or locale (e.g. Dublin-Belfast economic corridor)*’. This example refers to a report about spatial planning policy following the Brexit referendum and the Northern Ireland protocol that is unlikely to be broadly applicable. We have also suggested in the discussion section that in the future, the grey literature could help ‘*to better understand variation in implementation at scale, including evidence from specific locales in a geographically or politically bounded jurisdiction*.’

We agree that searching for more reviews and potentially gathering primary data from specific locales would be a useful next step, but based on the findings of the scoping review, that search would have to be geographically bounded for a comprehensive review of evidence from specific locales to be feasible.

**• Lines 203-204: “In the absence of information, we used consistent decision rules to assign role types based on policy instrument and general intervention types.” I think it would be more accurate to note this information as missing when it was missing, as the goal of the scoping review is to describe the evidence. Assuming actor roles doesn’t make sense to me in this context. Can the authors explain?**

As noted above, we have added four new columns to the data charting file with explicitly stated actor roles, conducted additional analyses and QA and clarified our approach and rationale in the methods section:

*‘We used consistent decision rules to identify which roles apply to each policy instrument to distinguish roles that may not be applicable to a particular instrument from roles where the actor information was missing. For example, regulation should always have an actor in an authorisation role, whereas grants should always have actors in financing and implementing roles. When the data extract from the review article did not specify actor roles, we updated roles in three circumstances, based on supplementary information: when the intervention was a named programme (e.g. Disabled Facilities Grant, Warm Homes programme), using information provided on their websites; for interventions where actor information was included in another data extract for the same specific intervention in the same country (e.g. Renters Rights Bill in the UK); and when interventions reflected duties that are the responsibility of a specific level of government (e.g. central government authorising changes to Capital Gains Tax and local authorities authorising local planning processes). The data charting file includes columns for the original, explicitly stated actor roles or lack thereof, as well as columns for updated roles based on this supplementary information, to enable decision-makers and practitioners to identify potential interventions they could use.’*

**Results:**
**• Suggest referring to the question # as you report the results.**
**• Given the contribution of this review to identifying evidence gaps, it would be helpful to provide a table or figure of those gaps that the reader could easily reference.**

We have updated the text to refer to research question numbers in the results and discussion sections. As noted above, we have also added a new subsection on Evidence gaps, including a new table to highlight this information.

Discussion:
• Well-written and helpful

**Reviewer #2**: This paper addresses a critical gap in housing and health research, and the inclusion of grey literature adds methodological novelty. However, the execution reveals a fundamental disconnect between the stated aim and the actual content: while the title claims to examine interventions that improve health, the manuscript largely stops at a taxonomy of housing policy instruments, with the health dimension nearly absent from the core analyses. The key shortcomings are detailed below, with corresponding recommendations.

**1. The concept of "health" is left undefined, and health measurement is absent.**
Although "improve health" is the stated goal, the manuscript provides no operational definition of health, nor does it specify the dimensions or measurement indicators of health outcomes. When discussing intervention effects, the text devotes substantial space to intermediate indicators—such as the number of physical modifications completed, rent changes, and market transactions—while content addressing actual health endpoints (e.g., mental health status, respiratory disease) is minimal and largely drawn from qualitative summaries of prior reviews without independent extraction or systematic analysis.

**Recommendation: The Methods section must include an operational definition of health outcomes and distinguish interventions that report health endpoints from those reporting only intermediate indicators, presenting them in stratified form in the Results. If health data are lacking for most interventions, the Discussion should candidly acknowledge this evidence gap rather than insinuate health effects from intermediate indicators in administrative reports.**

As noted in the response to the Editor, we have updated the abstract, introduction, methods, results and discussion sections (specific statements presented above), including a new table and analyses on types of effects, a new ‘Evidence gaps’ subsection and a more explicit discussion of the limited evidence on health effects. The updated data dictionary in the data charting file also provides specific examples of each effect type.

**2. The causal chain** is broken, and surrogate outcome fallacy is present.
The manuscript establishes no theoretical pathway by which housing interventions are expected to translate into health improvements, nor does it distinguish between the "intervention → change in housing condition" link and the "change in housing condition → health improvement" link. In multiple instances, "modifications completed" or "rent increases reduced" are treated as equivalent to health improvement, constituting a surrogate outcome fallacy. Moreover, 68% of included interventions lack any outcome data, yet are still discussed as part of the "evidence base"—a position that is logically untenable.

**Recommendation: Construct a layered analytical framework of "intervention → intermediate housing outcome → final health outcome" that explicitly signals where the current evidence stops. Add an evidence-strength classification, and adjust the conclusion's tone to a more cautious formulation, such as "identifies policy options with potential health implications, though their health effects remain largely untested."**

Analytical framework. As noted in the response to the Editor, in addition to citing existing conceptual frameworks in the introduction, we provide examples of pathways presented in two recent frameworks. The new table on types of effects indicates the range and frequency of reported evidence by type. The new ‘Evidence gaps’ subsection and new table discuss this limitation in greater depth in six paragraphs, including *‘When intervention effects were reported, they were often limited to shorter-term, proximate changes. The evidence base would benefit from more comprehensive assessments that track intervention activities and outputs through to interim outcomes, unintended consequences, effects on health conditions and population-level inequalities, particularly for interventions where causal pathways are less direct. Existing conceptual frameworks offer a structure through which this work could be undertaken [5,7–12].’*

Evidence strength classification. The data charting file includes information about the type of evidence and methods. The ‘Effectiveness of housing interventions’ subsection indicates ‘*The reviews included evaluations, surveys, case studies, statistical analyses, modelling, expert consultation, government policy guidance and evidence reviews; unfortunately, the level of variation and methodological detail means that were are not able to calculate the proportion of interventions that could be considered to have been robustly evaluated*.’ We have also added to the limitations section ‘*Furthermore, scoping reviews do not systematically assess study quality, so we are unable to calculate the proportion of studies that were weak, moderate or strong to confirm or refute this concern about the variable quality of the grey literature*.’

In the abstract and discussion section we refer to the *potential* of housing policies to influence health and have tempered the scope of claims throughout (using terms ‘may’ and ‘could’), particularly the ‘Effectiveness of housing interventions’ subsection and new ‘Evidence gaps’ subsection, as noted above. In the discussion section we have explicitly stated ‘*There are notable evidence gaps that the review surfaces. In particular, the review provides limited evidence to answer our second and fourth research questions on the effectiveness of interventions, both overall and specifically related to health, as well as on intervention costs and savings*.’

In the limitations section, we have added *‘As noted, based on the evidence we assessed, this scoping review is unable to draw substantive conclusions about the effects and cost of most interventions, and to a certain extent, actor roles.*’

**3. The scope** is misplaced, resulting in a "housing-heavy, health-light" study.
The Introduction reviews the literature on housing-health connections at length, but the Results and Analysis quickly shift to a Salamon-based taxonomy of policy instruments, with core findings organized around contrasts such as "economic regulation vs. direct provision." Health appears only as a label. The manuscript thus reads more like a housing policy or public administration paper, revealing a disciplinary misalignment with its stated public health aim.

**Recommendation: Honestly recalibrate the study's positioning. If the existing evidence base is retained, consider revising the title to "Housing policy instruments with potential health implications." Reduce the detailed exposition of policy instrument taxonomy, add a dedicated section on "From housing to health: gaps in the evidence chain," and discuss how future research might bridge the identified break.**

We agree with the reviewer’s characterisation of our results as ‘housing heavy, health light’ and drawing on a policy lens, and have made this intention more explicit in the discussion section *‘It bridges housing and health disciplines and research, policy and practice by more explicitly incorporating policy typologies and investigating key implementation considerations for research recommendations to be actionable. This is intended to better balance the literature, which has thus far been heavily oriented towards downstream health effects of exposures, with insufficient attention to the range of interventions and aspects of implementation that may be required to address housing conditions that in turn, could affect health.*’

As noted, we have added a new ‘Evidence gaps’ subsection and table, as suggested. Following the advice of the Editor, we have not changed the title of the scoping review.

**4. Grey literature bias** is not critically reflected upon.
The inclusion of grey literature is a distinctive feature, but the manuscript lacks a critical examination of its structural biases. Such literature tends to originate from administrative and advocacy bodies, prioritizing policy calls and experiential description over rigorous health outcome evaluation. This likely constitutes a key reason why 68% of interventions lack outcome data—a point the manuscript should address rather than simply treat as an observation.

**Recommendation: Add a critical discussion of grey literature source biases in the Limitations section and propose how future research might use leads from the grey literature to retrieve effect evidence from academic databases.**

As noted in the response to the Editor, we have been more explicit on grey literature bias in the discussion and limitations sections: ‘*These gaps are influenced in part by study limitations, as discussed below, and can be used to guide future research. … Our selection of evidence reviews, therefore, may help to explain why reviews of the majority of specific interventions did not report evidence on outcomes, costs or savings. The more rapid nature of reports in the grey literature may preclude measurement along multiple stages in a longer pathway of change and of more distal outcomes. An absent or less stringent peer review process may not require these types of outcomes as a condition of publication. These characteristics reflect longstanding critiques of the grey literature and the range of intended purposes, audiences, report formats and level of detail that they provide [116]. Furthermore, scoping reviews do not systematically assess study quality, so we are unable to calculate the proportion of studies that were weak, moderate or strong to confirm or refute this concern about the variable quality of the grey literature.’*

In the discussion section, we have added ‘*It also suggests ways that the grey and academic literatures could better be sequenced to inform one another. For example, the grey literature could be more substantively included in evidence reviews to identify an initial set of interventions and direct future academic research, focused on longer-term effects in order to guide decisions about targeting and expansion. The grey literature could then be subsequently used to better understand variation in implementation at scale, including evidence from specific locales in a geographically or politically bounded jurisdiction. Both the grey and academic literatures currently lack the level of detail on actor roles and costs needed for decision-makers, an important consideration for future work.*’

**5. Cost information** is fragmented and poorly presented.
Although the manuscript notes that cost data are scarce and highly variable, the presentation remains descriptive and unstructured, offering limited practical value for decision-makers.

**Recommendation: Include a summary table for interventions reporting cost data, specifying at least total investment, unit cost, funding source, and whether cost-saving analyses exist. Expand the Discussion to explore the policy implications of the "mismatch between who finances, implements, benefits, and saves."**

Summary table on costs. We agree that existing data on costs offers limited practical value for decision-makers and that information on total investment, unit cost, funding source and cost-savings analyses would be useful to summarise, if available. The data charting file includes the information we were able to extract on costs, but as noted in the response to the Editor, after reviewing the information on costs again and based on the limited (18% of interventions – the most incomplete of all of our data charting categories) and variable types of financial information (more so than interventions effects), we have not added a new cost summary table. In our judgment, a table would give the information more prominence than the data warrants. We have made that limitation clear in the abstract, results and discussion sections.

Policy implications of mismatch. We have added to the results section ‘*More transparent, robust comparisons, including cost-effectiveness and benefit-cost analyses would be a substantial contribution to the evidence base. Furthermore, until pooled budgets and substantive financial transfers between institutions are feasible (e.g. investing NHS savings to build or improve social housing), estimating costs and savings within the same institution (e.g. investing savings in local authority Adult Social Care costs into local authority housing interventions) will provide decision-makers with more actionable evidence*.’

In the discussion section we also highlight ‘*Both the grey and academic literatures currently lack the level of detail on actor roles and costs needed for decision-makers, an important improvement needed for future work. Understanding how costs and savings are unevenly distributed across actors may help to generate alternative financing options and move discussions beyond repeated calls for some actors to invest in housing improvements (e.g. social and private sector landlords), while savings accrue to others (e.g. NHS).*
