# Supplementary material for: Policy interventions to improve health through housing: A scoping review
Source: PLoS One. 2026 Sep 29;21(9):e0358689. doi: 10.1371/journal.pone.0358689 (PMC13623142; doi:10.1371/journal.pone.0358689)
Supplement: Supplementary file 3 [file pone.0358689.s005.docx]

We appreciate the careful attention to detail and specific suggestions recommended by the Editor. We have revised the methods and results sections, added a new table and updated the data charting file.

Below we provide a point-by-point response to each comment.

**Additional Editor Comments:
The revision is substantively responsive and the manuscript is much improved. I recommend the following changes:**

**1. Operational definition of health. Add brief Methods definitions of health status, self-reported health, and health/social care use endpoints, consistent with the S2 data dictionary. Revise the Study Selection statement that no restrictions were placed on outcome definitions so that it is clear that outcomes were not restricted by definition, but are nevertheless operationalised for this review.**

We have updated the following Methods subsections as follows:

Study Selection: ‘We did not place restrictions on geography, the type of effects reported or how outcomes related to health were defined, instead documenting and classifying existing evidence to clarify the boundaries of this topic area, as scoping reviews are intended to do. *We therefore included studies reporting any type of effect, including but not limited to health or a specific definition of health, and subsequently categorised type of effect in the data charting stage*.’

Data Charting: *‘The data dictionary provides explanations and examples for each effect type. For example, health status covers both physical and mental health and includes specific conditions (e.g. asthma), injuries, life expectancy and functional ability. Self-reported/perceived health covers physical, mental and general health, as reported by study respondents, perceived quality of life, wellbeing and safety. General health is a self-reported question in surveys designed by the UK Office for National Statistics which asks respondents to rate on a 5-point scale from very good to very bad ‘How is your health in general?’ Health or social care use includes hospital admissions, falls requiring medical attention and hours of home care.’*

**2. Show where the evidence reaches. Table 2 aggregates effect types but does not identify which interventions reach a health endpoint. As requested in the previous decision, add a cross-tabulation of intervention/policy type against a tiered outcome classification: health endpoint; intermediate housing/market/economic outcome; output/process only; no effect reported.**

We have added a new table (#3) that presents a cross-tabulation of effect type and policy instruments and added the following text: *‘Looking across policy instruments, more health-related outcomes were reported for direct or contracted provision of goods and services – albeit still a small proportion of all interventions (n=31; 8%) (Table 3). Other intermediate outcomes were more common for fiscal policy and economic regulation (n=33; 8%).’*

The subsequent five paragraphs present results in greater depth for specific interventions, including examples of the types of effects that have been reported.

**3. Reconcile reported numbers with the S2 file. Please check and correct the manuscript and tables, including: social regulation (80 vs 81); housing-domain percentages and denominators; the cost count (63/18%); actor-role missingness percentages; and the Decent Homes Programme funding figure (£22bn + £15bn, not £22bn + £37bn).**

Thank you for bringing a fresh set of eyes to help catch the social regulation and Decent Homes typos, which we have corrected. We have also recalculated and updated the text to further specify housing domain and cost percentages and denominators.

The previous statement about missing information on actor roles was based on counts of individual actors so we have changed how that information is presented so it is consistent with other calculations and simpler to reproduce, using all records (n=412) and calculating the proportion of applicable roles that were not stated. The revised text includes percentages, numerators and denominators since the proportion of applicable roles varies by policy instrument (e.g. enforcement roles are less common than other roles). As part of these updates, we conducted an additional data quality check on cell values, confirmed the counts in Table 4 (actor roles), total number of actor types and proportion of unique interventions that involved more than one actor.

**4. Define the analytic bases. Table 1 is based on 327 unique interventions, whereas Tables 2–3 use 412 records. State this explicitly in the Methods.**

In the Collating, Summarising and Reporting Results subsection, we have added*: ‘We present findings as a proportion of: all reported interventions (n=412), unique interventions (n=327) and interventions reporting any type of effect (n=132). Table 1 (frequency of policy instrument and general intervention type) and text presenting frequency of housing domains use unique interventions as the denominator, since multiple studies report on the same intervention. Table 2 (types of effects) presents the proportion of interventions reporting any type of effect. Table 3 (type of effect by policy instrument) and text presenting effectiveness of interventions and costs use all interventions as the denominator, since different studies reporting on the same intervention may report different effects or costs. Table 4 (actor roles) also draws from all reported interventions for the same reason and presents the total number of times a specific actor was involved in a specific role.’*

We have also added footnotes to:

Table 2: ‘*More than one third of interventions reporting any effects mentioned more than one type of effect (n=47/132; 36%), reflected in the count total*.’

Table 4: *‘Table totals do not sum to 412 because not all roles are applicable to all policy instruments, more than one actor type may be involved in the same role, particularly implementation, and infrequently mentioned actor types listed below are not presented in the table.’*

Throughout the results section, we have further specified all or unique interventions in the text and added numerators and denominators, so calculations are more easily reproducible.

**5. Distinguish reported from inferred actor roles. For Table 3, show the as-reported figures alongside the updated figures, or clearly indicate in the table/footnote the proportion of classifications derived from supplementary sources. Improve search reproducibility.**

In the table on actor roles (now #4), we have added a footnote ‘*Across all interventions (n=412), unstated actor roles were updated based on supplementary information for 42 (10%) interventions regarding authorising roles, 133 (32%) regarding financing roles, 117 (28%) regarding implementing roles and 30 (7%) regarding enforcing roles. Both original and updated actor roles are included in the Data Charting File.’*
