# Supplementary material for: Policy interventions to improve health through housing: A scoping review
Source: PLoS One. 2026 Sep 29;21(9):e0358689. doi: 10.1371/journal.pone.0358689 (PMC13623142; doi:10.1371/journal.pone.0358689)
Supplement: Peer review history

# Peer review: Decision Letter 0

**DOI:** 10.1371/journal.pone.0358689.r001

**Authors:** Zhou Yu

20 May 2026

PONE-D-26-07288

Policy interventions to improve health through housing: A scoping review

PLOS One

Dear Dr. Buffardi,

Thank you for submitting your manuscript to PLOS ONE. After careful consideration, we feel that it has merit but does not fully meet PLOS ONE’s publication criteria as it currently stands. Therefore, we invite you to submit a revised version of the manuscript that addresses the points raised during the review process.

==============================

Both reviewers recommend major revision, which I support. The manuscript addresses an important question and the data charting file is a valuable resource, but several issues must be resolved before the conclusions are adequately supported by the data, per PLOS ONE's publication criteria.

Required for acceptance

Operationalise "health" and stratify by outcome type. Health is never defined, and the Results slide between intervention completion, housing change, and health outcomes (Reviewer 2). The Methods must specify what counts as a health endpoint, and the Results must distinguish interventions reporting health endpoints from those reporting only intermediate measures. A layered framework, signposting where evidence stops, would address this without requiring the retitle Reviewer 2 suggests.

Tighten inclusion criteria and the scope of claims. State the source-level information required for an intervention to be included and analysed. Many reports lack the granularity to support claims about effectiveness, cost, or actor roles. Two specific implications: (a) the actor-role imputation flagged by Reviewer 1 (lines 203–204) should be reported as missing rather than filled by decision rule; and (b) the use of health-system funding to support housing interventions is a worthwhile research direction but one this corpus cannot adequately answer, and should be framed carefully or deferred to future work.

Reflect critically on grey literature bias. The 68% no-outcome figure is partly an artifact of source selection (Reviewer 2). Limitations should address this and indicate how future research might use grey literature leads to retrieve effect evidence from academic databases.

Recommended

An evidence gaps table (R1) and a cost-summary table (R2) would strengthen the resource value.

Number the four research questions and reference them through the Results; justify the locale exclusion more fully (R1).

One framing point. AI tools can now generate intervention inventories quickly but cannot guarantee provenance, defensible framing, or practitioner relevance. The distinctive value of a scoping review like this lies in curation, judgment, and the auditable data charting file — a sentence to this effect in the Discussion might help readers locate the contribution.

Reconciling the reviews. Reviewer 1's points are worth addressing as written. Reviewer 2's framework critique should be taken seriously, but does not require retitling; the required changes above are necessary.

==============================

Please submit your revised manuscript by Jul 04 2026 11:59PM. If you will need more time than this to complete your revisions, please reply to this message or contact the journal office at plosone@plos.org. When you're ready to submit your revision, log on to https://www.editorialmanager.com/pone/ and select the 'Submissions Needing Revision' folder to locate your manuscript file.

Please include the following items when submitting your revised manuscript:

- A letter that responds to each point raised by the academic editor and reviewer(s). You should upload this letter as a separate file labeled 'Response to Reviewers'.
- A marked-up copy of your manuscript that highlights changes made to the original version. You should upload this as a separate file labeled 'Revised Manuscript with Track Changes'.
- An unmarked version of your revised paper without tracked changes. You should upload this as a separate file labeled 'Manuscript'.

If you would like to make changes to your financial disclosure, please include your updated statement in your cover letter. Guidelines for resubmitting your figure files are available below the reviewer comments at the end of this letter.

If applicable, we recommend that you deposit your laboratory protocols in protocols.io to enhance the reproducibility of your results. Protocols.io assigns your protocol its own identifier (DOI) so that it can be cited independently in the future. For instructions see: https://journals.plos.org/plosone/s/submission-guidelines#loc-laboratory-protocols. Additionally, PLOS ONE offers an option for publishing peer-reviewed Lab Protocol articles, which describe protocols hosted on protocols.io. Read more information on sharing protocols at https://plos.org/protocols?utm_medium=editorial-email&utm_source=authorletters&utm_campaign=protocols.

As the corresponding author, your ORCID iD is verified in the submission system and will appear in the published article. PLOS supports the use of ORCID, and we encourage all coauthors to register for an ORCID iD and use it as well. Please encourage your coauthors to verify their ORCID iD within the submission system before final acceptance, as unverified ORCID iDs will not appear in the published article. Only the individual author can complete the verification step; PLOS staff cannot verify ORCID iDs on behalf of authors.

We look forward to receiving your revised manuscript.

Kind regards,

Zhou Yu, PhD

Academic Editor

PLOS One

When submitting your revision, we need you to address these additional requirements.

1. Please ensure that your manuscript meets PLOS ONE's style requirements, including those for file naming. The PLOS ONE style templates can be found at

https://journals.plos.org/plosone/s/file?id=wjVg/PLOSOne_formatting_sample_main_body.pdf and

https://journals.plos.org/plosone/s/file?id=ba62/PLOSOne_formatting_sample_title_authors_affiliations.pdf

2. If the reviewer comments include a recommendation to cite specific previously published works, please review and evaluate these publications to determine whether they are relevant and should be cited. There is no requirement to cite these works unless the editor has indicated otherwise.

[Note: HTML markup is below. Please do not edit.]

Reviewers' comments:

Reviewer's Responses to Questions

**Comments to the Author**

1. Is the manuscript technically sound, and do the data support the conclusions?

Reviewer #1: Partly

Reviewer #2: Partly

**********

2. Has the statistical analysis been performed appropriately and rigorously?

Reviewer #1: N/A

Reviewer #2: Yes

**********

3. Have the authors made all data underlying the findings in their manuscript fully available?

The PLOS Data policy

Reviewer #1: Yes

Reviewer #2: Yes

**********

4. Is the manuscript presented in an intelligible fashion and written in standard English?

Reviewer #1: Yes

Reviewer #2: Yes

**********

Reviewer #1: Thank you for the opportunity to review this paper, which is a helpful scoping review of housing interventions to improve public health. Overall I think the idea and implementation of this project are strong, but I have a few methodological concerns along with some small suggestions on paper presentation. Please see full comments below.

Abstract:

• Abstract is clear

Introduction:

• Helpful framing

• Typo, line 126: should be “determine IF a full systematic review is warranted”

• Would be helpful to number the four questions so they can be easily referenced later

Methods:

• It seems like there would be a lot of evidence from specific locales, so it’s unclear to me why these were excluded from the current review. Can the authors provide more justification for this decision? Why is this a review of reviews rather than an overall review of the evidence?

• Lines 203-204: “In the absense of information, we used consistent decision rules to assign role types based on policy instrument and general intervention types.” I think it would be more accurate to note this information as missing when it was missing, as the goal of the scoping review is to describe the evidence. Assuming actor roles doesn’t make sense to me in this context. Can the authors explain?

Results:

• Suggest referring to the question # as you report the results.

• Given the contribution of this review to identifying evidence gaps, it would be helpful to provide a table or figure of those gaps that the reader could easily reference.

Discussion:

• Well-written and helpful

Reviewer #2: This paper addresses a critical gap in housing and health research, and the inclusion of grey literature adds methodological novelty. However, the execution reveals a fundamental disconnect between the stated aim and the actual content: while the title claims to examine interventions that improve health, the manuscript largely stops at a taxonomy of housing policy instruments, with the health dimension nearly absent from the core analyses. The key shortcomings are detailed below, with corresponding recommendations.

1. The concept of "health" is left undefined, and health measurement is absent.

Although "improve health" is the stated goal, the manuscript provides no operational definition of health, nor does it specify the dimensions or measurement indicators of health outcomes. When discussing intervention effects, the text devotes substantial space to intermediate indicators—such as the number of physical modifications completed, rent changes, and market transactions—while content addressing actual health endpoints (e.g., mental health status, respiratory disease) is minimal and largely drawn from qualitative summaries of prior reviews without independent extraction or systematic analysis.

Recommendation: The Methods section must include an operational definition of health outcomes and distinguish interventions that report health endpoints from those reporting only intermediate indicators, presenting them in stratified form in the Results. If health data are lacking for most interventions, the Discussion should candidly acknowledge this evidence gap rather than insinuate health effects from intermediate indicators in administrative reports.

2. The causal chain is broken, and surrogate outcome fallacy is present.

The manuscript establishes no theoretical pathway by which housing interventions are expected to translate into health improvements, nor does it distinguish between the "intervention → change in housing condition" link and the "change in housing condition → health improvement" link. In multiple instances, "modifications completed" or "rent increases reduced" are treated as equivalent to health improvement, constituting a surrogate outcome fallacy. Moreover, 68% of included interventions lack any outcome data, yet are still discussed as part of the "evidence base"—a position that is logically untenable.

Recommendation: Construct a layered analytical framework of "intervention → intermediate housing outcome → final health outcome" that explicitly signals where the current evidence stops. Add an evidence-strength classification, and adjust the conclusion's tone to a more cautious formulation, such as "identifies policy options with potential health implications, though their health effects remain largely untested."

3. The scope is misplaced, resulting in a "housing-heavy, health-light" study.

The Introduction reviews the literature on housing-health connections at length, but the Results and Analysis quickly shift to a Salamon-based taxonomy of policy instruments, with core findings organized around contrasts such as "economic regulation vs. direct provision." Health appears only as a label. The manuscript thus reads more like a housing policy or public administration paper, revealing a disciplinary misalignment with its stated public health aim.

Recommendation: Honestly recalibrate the study's positioning. If the existing evidence base is retained, consider revising the title to "Housing policy instruments with potential health implications." Reduce the detailed exposition of policy instrument taxonomy, add a dedicated section on "From housing to health: gaps in the evidence chain," and discuss how future research might bridge the identified break.

4. Grey literature bias is not critically reflected upon.

The inclusion of grey literature is a distinctive feature, but the manuscript lacks a critical examination of its structural biases. Such literature tends to originate from administrative and advocacy bodies, prioritizing policy calls and experiential description over rigorous health outcome evaluation. This likely constitutes a key reason why 68% of interventions lack outcome data—a point the manuscript should address rather than simply treat as an observation.

Recommendation: Add a critical discussion of grey literature source biases in the Limitations section, and propose how future research might use leads from the grey literature to retrieve effect evidence from academic databases.

5. Cost information is fragmented and poorly presented.

Although the manuscript notes that cost data are scarce and highly variable, the presentation remains descriptive and unstructured, offering limited practical value for decision-makers.

Recommendation: Include a summary table for interventions reporting cost data, specifying at least total investment, unit cost, funding source, and whether cost-saving analyses exist. Expand the Discussion to explore the policy implications of the "mismatch between who finances, implements, benefits, and saves."

The shortcomings identified above—particularly the undefined concept of health and the broken causal chain—reflect fundamental deficits in the research framework rather than issues resolvable by minor revision. That said, given the value of the topic and the exploratory contribution of the grey literature approach, I recommend Major Revision and Re-review. The authors should submit a point-by-point response addressing the above concerns and include a revised analytic framework diagram to clarify the revised logic of the manuscript.

**********

what does this mean?). If published, this will include your full peer review and any attached files.

If you choose “no”, your identity will remain anonymous but your review may still be made public.

**Do you want your identity to be public for this peer review?**  For information about this choice, including consent withdrawal, please see our Privacy Policy

Reviewer #1: No

Reviewer #2: No

**********

[NOTE: If reviewer comments were submitted as an attachment file, they will be attached to this email and accessible via the submission site. Please log into your account, locate the manuscript record, and check for the action link "View Attachments". If this link does not appear, there are no attachment files.]

To ensure your figures meet our technical requirements, please review our figure guidelines: https://journals.plos.org/plosone/s/figures

You may also use PLOS’s free figure tool, NAAS, to help you prepare publication quality figures: https://journals.plos.org/plosone/s/figures#loc-tools-for-figure-preparation.

NAAS will assess whether your figures meet our technical requirements by comparing each figure against our figure specifications.

# Author comment: Author response to Decision Letter 1

**DOI:** 10.1371/journal.pone.0358689.r002

7 Jul 2026

We thank the reviewers for their detailed and helpful questions and recommendations and the editor for the very useful clarification on revisions and reconciling reviewers’ comments.

We have revised the abstract, introduction, methods, results and discussion sections and added 2 new tables to the manuscript. We have also updated the data dictionary and added 6 new columns to the data charting file.

The attached 10 page response to reviewers document provides a point-by-point response to each comment.

Attachment Submitted filename: response to reviewers.docx

# Editor report: Decision Letter 1

**DOI:** 10.1371/journal.pone.0358689.r003

**Authors:** Zhou Yu

24 Aug 2026

Dear Dr. Buffardi,

Thank you for submitting your manuscript to PLOS One. After careful consideration, we feel that it has merit but does not fully meet PLOS One’s publication criteria as it currently stands. Therefore, we invite you to submit a revised version of the manuscript that addresses the points raised during the review process.

Please submit your revised manuscript by Oct 08 2026 11:59PM. If you will need more time than this to complete your revisions, please reply to this message or contact the journal office at plosone@plos.org. When you're ready to submit your revision, log on to https://www.editorialmanager.com/pone/ and select the 'Submissions Needing Revision' folder to locate your manuscript file.

- A letter that responds to each point raised by the academic editor and reviewer(s). You should upload this letter as a separate file labeled 'Response to Reviewers'.
- A marked-up copy of your manuscript that highlights changes made to the original version. You should upload this as a separate file labeled 'Revised Manuscript with Track Changes'.
- An unmarked version of your revised paper without tracked changes. You should upload this as a separate file labeled 'Manuscript'.

If you would like to make changes to your financial disclosure, please include your updated statement in your cover letter. Guidelines for resubmitting your figure files are available below the reviewer comments at the end of this letter.

If applicable, we recommend that you deposit your laboratory protocols in protocols.io to enhance the reproducibility of your results. Protocols.io assigns your protocol its own identifier (DOI) so that it can be cited independently in the future. For instructions see: https://journals.plos.org/plosone/s/submission-guidelines#loc-laboratory-protocols. Additionally, PLOS One offers an option for publishing peer-reviewed Lab Protocol articles, which describe protocols hosted on protocols.io. Read more information on sharing protocols at https://plos.org/protocols?utm_medium=editorial-email&utm_source=authorletters&utm_campaign=protocols.

As the corresponding author, your ORCID iD is verified in the submission system and will appear in the published article. PLOS supports the use of ORCID, and we encourage all coauthors to register for an ORCID iD and use it as well. Please encourage your coauthors to verify their ORCID iD within the submission system before final acceptance, as unverified ORCID iDs will not appear in the published article. *Only*  the individual author can complete the verification step; PLOS staff *cannot*  verify ORCID iDs on behalf of authors.

We look forward to receiving your revised manuscript.

Kind regards,

Zhou Yu, PhD

Academic Editor

PLOS One

Journal Requirements:

If the reviewer comments include a recommendation to cite specific previously published works, please review and evaluate these publications to determine whether they are relevant and should be cited. There is no requirement to cite these works unless the editor has indicated otherwise.

Please review your reference list to ensure that it is complete and correct. If you have cited papers that have been retracted, please include the rationale for doing so in the manuscript text, or remove these references and replace them with relevant current references. Any changes to the reference list should be mentioned in the rebuttal letter that accompanies your revised manuscript. If you need to cite a retracted article, indicate the article’s retracted status in the References list and also include a citation and full reference for the retraction notice.

Additional Editor Comments:

The revision is substantively responsive and the manuscript is much improved. I recommend the following changes:

1. Operational definition of health. Add brief Methods definitions of health status, self-reported health, and health/social care use endpoints, consistent with the S2 data dictionary. Revise the Study Selection statement that no restrictions were placed on outcome definitions so that it is clear that outcomes were not restricted by definition, but are nevertheless operationalised for this review.

2. Show where the evidence reaches. Table 2 aggregates effect types but does not identify which interventions reach a health endpoint. As requested in the previous decision, add a cross-tabulation of intervention/policy type against a tiered outcome classification: health endpoint; intermediate housing/market/economic outcome; output/process only; no effect reported.

3. Reconcile reported numbers with the S2 file. Please check and correct the manuscript and tables, including: social regulation (80 vs 81); housing-domain percentages and denominators; the cost count (63/18%); actor-role missingness percentages; and the Decent Homes Programme funding figure (£22bn + £15bn, not £22bn + £37bn).

4. Define the analytic bases. Table 1 is based on 327 unique interventions, whereas Tables 2–3 use 412 records. State this explicitly in the Methods.

5. Distinguish reported from inferred actor roles. For Table 3, show the as-reported figures alongside the updated figures, or clearly indicate in the table/footnote the proportion of classifications derived from supplementary sources.

Improve search reproducibility.

[Note: HTML markup is below. Please do not edit.]

[NOTE: If reviewer comments were submitted as an attachment file, they will be attached to this email and accessible via the submission site. Please log into your account, locate the manuscript record, and check for the action link "View Attachments". If this link does not appear, there are no attachment files.]

To ensure your figures meet our technical requirements, please review our figure guidelines: https://journals.plos.org/plosone/s/figures

You may also use PLOS’s free figure tool, NAAS, to help you prepare publication quality figures: https://journals.plos.org/plosone/s/figures#loc-tools-for-figure-preparation.

NAAS will assess whether your figures meet our technical requirements by comparing each figure against our figure specifications.

# Author comment: Author response to Decision Letter 2

**DOI:** 10.1371/journal.pone.0358689.r004

2 Sep 2026

We appreciate the careful attention to detail and specific suggestions recommended by the Editor. We have revised the methods and results sections, added a new table and updated the data charting file.

Below we provide a point-by-point response to each comment.

Additional Editor Comments:

The revision is substantively responsive and the manuscript is much improved. I recommend the following changes:

1. Operational definition of health. Add brief Methods definitions of health status, self-reported health, and health/social care use endpoints, consistent with the S2 data dictionary. Revise the Study Selection statement that no restrictions were placed on outcome definitions so that it is clear that outcomes were not restricted by definition, but are nevertheless operationalised for this review.

We have updated the following Methods subsections as follows:

Study Selection: ‘We did not place restrictions on geography, the type of effects reported or how outcomes related to health were defined, instead documenting and classifying existing evidence to clarify the boundaries of this topic area, as scoping reviews are intended to do. We therefore included studies reporting any type of effect, including but not limited to health or a specific definition of health, and subsequently categorised type of effect in the data charting stage.’

Data Charting: ‘The data dictionary provides explanations and examples for each effect type. For example, health status covers both physical and mental health and includes specific conditions (e.g. asthma), injuries, life expectancy and functional ability. Self-reported/perceived health covers physical, mental and general health, as reported by study respondents, perceived quality of life, wellbeing and safety. General health is a self-reported question in surveys designed by the UK Office for National Statistics which asks respondents to rate on a 5-point scale from very good to very bad ‘How is your health in general?’ Health or social care use includes hospital admissions, falls requiring medical attention and hours of home care.’

2. Show where the evidence reaches. Table 2 aggregates effect types but does not identify which interventions reach a health endpoint. As requested in the previous decision, add a cross-tabulation of intervention/policy type against a tiered outcome classification: health endpoint; intermediate housing/market/economic outcome; output/process only; no effect reported.

We have added a new table (#3) that presents a cross-tabulation of effect type and policy instruments and added the following text: ‘Looking across policy instruments, more health-related outcomes were reported for direct or contracted provision of goods and services – albeit still a small proportion of all interventions (n=31; 8%) (Table 3). Other intermediate outcomes were more common for fiscal policy and economic regulation (n=33; 8%).’

The subsequent five paragraphs present results in greater depth for specific interventions, including examples of the types of effects that have been reported.

3. Reconcile reported numbers with the S2 file. Please check and correct the manuscript and tables, including: social regulation (80 vs 81); housing-domain percentages and denominators; the cost count (63/18%); actor-role missingness percentages; and the Decent Homes Programme funding figure (£22bn + £15bn, not £22bn + £37bn).

Thank you for bringing a fresh set of eyes to help catch the social regulation and Decent Homes typos, which we have corrected. We have also recalculated and updated the text to further specify housing domain and cost percentages and denominators.

The previous statement about missing information on actor roles was based on counts of individual actors so we have changed how that information is presented so it is consistent with other calculations and simpler to reproduce, using all records (n=412) and calculating the proportion of applicable roles that were not stated. The revised text includes percentages, numerators and denominators since the proportion of applicable roles varies by policy instrument (e.g. enforcement roles are less common than other roles). As part of these updates, we conducted an additional data quality check on cell values, confirmed the counts in Table 4 (actor roles), total number of actor types and proportion of unique interventions that involved more than one actor.

4. Define the analytic bases. Table 1 is based on 327 unique interventions, whereas Tables 2–3 use 412 records. State this explicitly in the Methods.

In the Collating, Summarising and Reporting Results subsection, we have added: ‘We present findings as a proportion of: all reported interventions (n=412), unique interventions (n=327) and interventions reporting any type of effect (n=132). Table 1 (frequency of policy instrument and general intervention type) and text presenting frequency of housing domains use unique interventions as the denominator, since multiple studies report on the same intervention. Table 2 (types of effects) presents the proportion of interventions reporting any type of effect. Table 3 (type of effect by policy instrument) and text presenting effectiveness of interventions and costs use all interventions as the denominator, since different studies reporting on the same intervention may report different effects or costs. Table 4 (actor roles) also draws from all reported interventions for the same reason and presents the total number of times a specific actor was involved in a specific role.’

We have also added footnotes to:

Table 2: ‘More than one third of interventions reporting any effects mentioned more than one type of effect (n=47/132; 36%), reflected in the count total.’

Table 4: ‘Table totals do not sum to 412 because not all roles are applicable to all policy instruments, more than one actor type may be involved in the same role, particularly implementation, and infrequently mentioned actor types listed below are not presented in the table.’

Throughout the results section, we have further specified all or unique interventions in the text and added numerators and denominators, so calculations are more easily reproducible.

5. Distinguish reported from inferred actor roles. For Table 3, show the as-reported figures alongside the updated figures, or clearly indicate in the table/footnote the proportion of classifications derived from supplementary sources. Improve search reproducibility.

In the table on actor roles (now #4), we have added a footnote ‘Across all interventions (n=412), unstated actor roles were updated based on supplementary information for 42 (10%) interventions regarding authorising roles, 133 (32%) regarding financing roles, 117 (28%) regarding implementing roles and 30 (7%) regarding enforcing roles. Both original and updated actor roles are included in the Data Charting File.’

Attachment Submitted filename: response to editor.docx

# Editor report: Decision Letter 2

**DOI:** 10.1371/journal.pone.0358689.r005

**Authors:** Zhou Yu

3 Sep 2026

Policy interventions to improve health through housing: A scoping review

PONE-D-26-07288R2

Dear Dr. Buffardi,

We’re pleased to inform you that your manuscript has been judged scientifically suitable for publication and will be formally accepted for publication once it meets all outstanding technical requirements.

Within one week, you’ll receive an e-mail detailing the required amendments. When these have been addressed, you’ll receive a formal acceptance letter and your manuscript will be scheduled for publication.

An invoice will be generated when your article is formally accepted. Please note, if your institution has a publishing partnership with PLOS and your article meets the relevant criteria, all or part of your publication costs will be covered. Please make sure your user information is up-to-date by logging into Editorial Manager at Editorial Manager® and clicking the ‘Update My Information' link at the top of the page. For questions related to billing, please contact billing support.

If your institution or institutions have a press office, please notify them about your upcoming paper to help maximize its impact. If they’ll be preparing press materials, please inform our press team as soon as possible -- no later than 48 hours after receiving the formal acceptance. Your manuscript will remain under strict press embargo until 2 pm Eastern Time on the date of publication. For more information, please contact onepress@plos.org.

Kind regards,

Zhou Yu, PhD

Academic Editor

PLOS One

Additional Editor Comments (optional):

There are a few minor issues. For example, “means that were are not able” (line 359) contains a typo. In addition, Table 3 should include within-row percentages, or the relevant percentages should be reported in the text.

Reviewers' comments:

# Editor report: Acceptance letter

**DOI:** 10.1371/journal.pone.0358689.r006

**Authors:** Zhou Yu

PONE-D-26-07288R2

PLOS One

Dear Dr. Buffardi,

I'm pleased to inform you that your manuscript has been deemed suitable for publication in PLOS One. Congratulations! Your manuscript is now being handed over to our production team.

At this stage, our production department will prepare your paper for publication. This includes ensuring the following:

* All references, tables, and figures are properly cited

* All relevant supporting information is included in the manuscript submission,

* There are no issues that prevent the paper from being properly typeset

You will receive further instructions from the production team, including instructions on how to review your proof when it is ready. Please keep in mind that we are working through a large volume of accepted articles, so please give us a few days to review your paper and let you know the next and final steps.

Lastly, if your institution or institutions have a press office, please let them know about your upcoming paper now to help maximize its impact. If they'll be preparing press materials, please inform our press team within the next 48 hours. Your manuscript will remain under strict press embargo until 2 pm Eastern Time on the date of publication. For more information, please contact onepress@plos.org.

You will receive an invoice from PLOS for your publication fee after your manuscript has reached the completed accept phase. If you receive an email requesting payment before acceptance or for any other service, this may be a phishing scheme. Learn how to identify phishing emails and protect your accounts at https://explore.plos.org/phishing.

If we can help with anything else, please email us at customercare@plos.org.

Thank you for submitting your work to PLOS One and supporting open access.

Kind regards,

PLOS One Editorial Office Staff

on behalf of

Dr. Zhou Yu

Academic Editor

PLOS One
